# A Novel Synthesis Process of Cholesterol Ester by the Cross-Coupling of Cholesterol and Aroyl Chlorides

**DOI:** 10.4236/ijoc.2024.144007

**Published:** 2024-12-03

**Authors:** Mohammad Al-Masum, Brooklyn Phillips

**Affiliations:** Department of Chemistry, Tennessee State University, Nashville, TN, USA

**Keywords:** Cholesterol, Cholesterol Esters, Cross-Coupling, Microwave, Aroyl Chloride

## Abstract

Cholesterol is a sterol with a four-membered ring structure and a single hydroxyl group attached to one of the rings. It is the active, raw form of cholesterol. Cholesteryl Ester is the inactive form in which cholesterol is esterified to be transported to target organs. The newly developed process of making bulky esters from unprecedented tertiary alcohols is applied to synthesize cholesterol esters successfully. Although cholesterol is a secondary alcohol, it is a very bulky and interesting compound due to its immense application. Usually, specific enzymes catalyze cholesterol to cholesterol ester. This project aims to develop a cross-coupling chemistry process to synthesize cholesterol esters and see the broader application of those new cholesterol esters in chemical biology. The mixture of cholesterol, sodium tert-butoxide, aroyl chloride, the catalyst PdCl_2_ (d^t^bpf) complex, and the solvent 1,4-dioxane, when microwaved at 100˚C for 2 hours, works well for the formation of cross-coupling cholesterol ester products in good to high yields.

## 1. Introduction

**Scheme 1.**
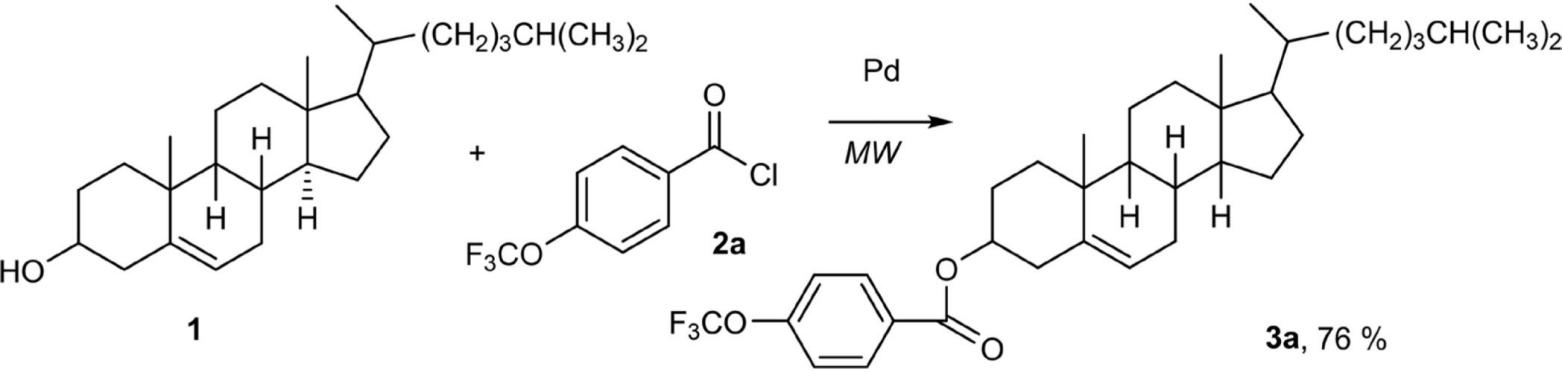
Cholesterol ester from cholesterol and aroyl chlorides.

Cholesterol alone is an essential compound for the human body. It is a structural component within the plasma membrane that provides stability and acts as the backbone of sex hormones (progesterone, androgen, testosterone, etc.), bile production, and overall cell building and repair [1]. However, when a fatty acid is attached to the hydroxyl group of cholesterol, it then yields cholesterol ester. Cholesterol esters function within the human body by behaving as a warehouse storing cholesterol and transporting it through the veins to the targeted parts of the body [2]. These specific esters are commonly found in the liver, plasma, and stored as lipid droplets within the human brain. Normally, biological systems catalyze cholesterol esters by the enzymes lecithin: cholesterol acyltransferase (LCAT) or acylcoenzyme A: cholesterol acyltransferase (ACAT). Whether the enzyme LCAT or ACAT is used depends on the location of the reaction’s synthesis. For example, LCAT catalyzed reactions would occur in peripheral tissues (i.e. skin, gut, lung) and for ACAT, it can vary between the intestine, liver, and endoplasmic reticulum (ER) [3]. However, this work aims to identify a novel way of synthesizing cholesterol esters, not by using biocatalyst, as done often, but by using cross-coupling chemistry. Cross-coupling chemistry gives an advantage because combining an aroyl chloride and cholesterol allows for a smoother building-up and breaking-down synthesis process (Scheme 1).

## 2. Results and Discussion

By using green chemistry, this study was able to reduce health hazards and substances along with limiting chemical pollutants. An alternative technology that was used was microwave chemistry.

**Figure 1.**
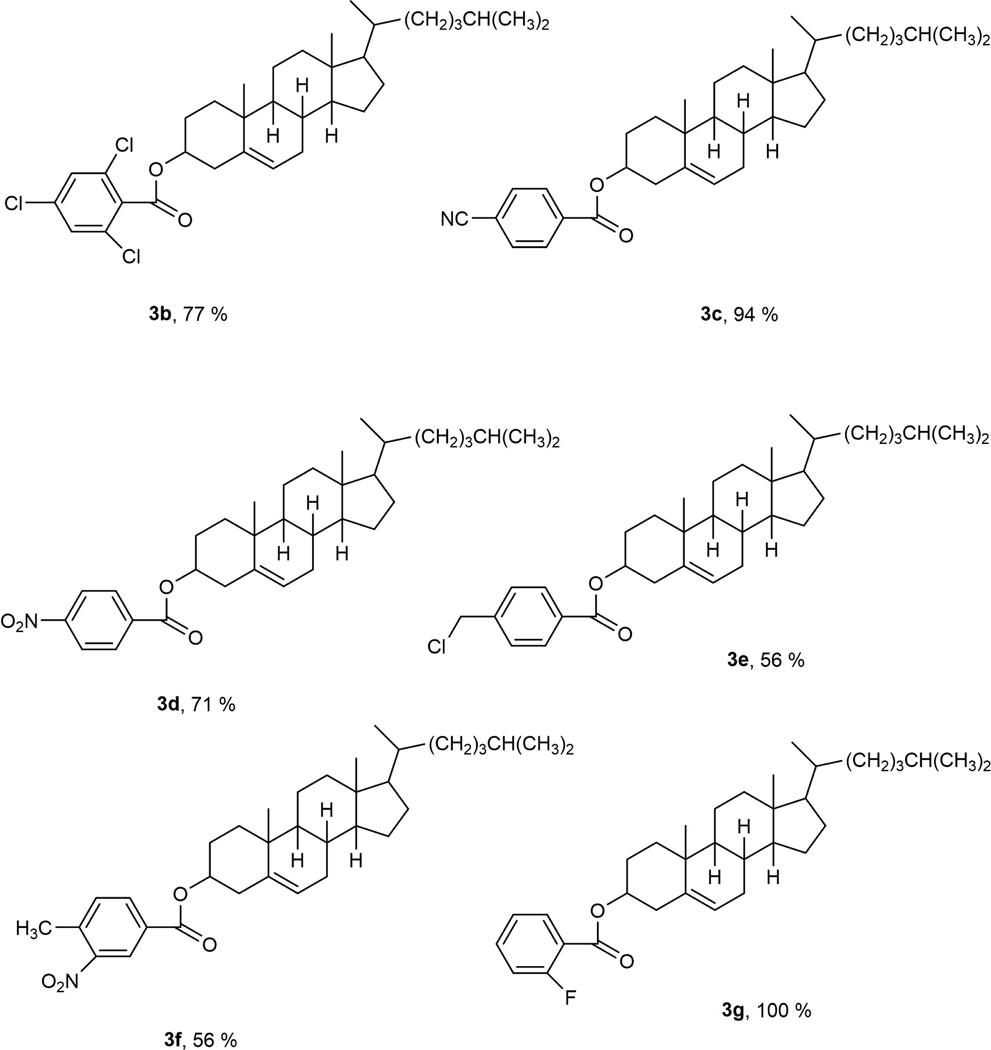
Cholesterol esters from aroyl chlorides 2b, 2c, 2d, 2e, 2f and 2g.

Microwave chemistry is an eco-friendlier mechanism than that of traditional approaches and creates a more energy efficient method for administering heat and consuming less energy [4]. Furthermore, when running this reaction, the base sodium tert-butoxide (NaOtBu), along with a palladium catalyst (PdCl_2_ (d^t^bpf)) was used to help develop this reaction. Palladium is a transition metal that is known for its victorious efforts in promoting the progress of compounds within palladium-catalyzed cross-coupling reactions [5] [6]. Throughout this experiment, we used various aroyl chlorides, different microwave temperatures, and different ratios of cholesterol to aroyl chloride. When analyzing the data to find the optimum condition for the best percent yield, it was noticeable that the reactions whose ratio was a 1 to 2, that being 0.5 mols of cholesterol and 1.0 mol of aroyl chloride, ran for 2 hours at 100˚C and 120˚C did better overall. The aroyl chlorides that were used to gain these newly found cholesterol esters were 4-trifluoromethoxy benzoyl chloride (**2a)**, 2,4,6-trichlorobenzoyl chloride (**2b)**, 4-cyanobenzoyl benzoyl chloride (**2c**), 4-nitrobenzoyl chloride **(2d)**, 4-(Chloromethyl) benzoyl chloride **(2e)**, 4-Methyl-3-nitrobenzoyl chloride **(2f)** and 2-fluorobenzoyl chloride (**2g**). Figure 1 shows the results of cholesterol esters in percentage yields (**3a**, 76 %), (**3b**, 77%), (**3c**, 94 %), (**3d**, 71%), (**3e**, 56%), (**3f**, 56%), and (**3g**, 100 %).

Cholesterol esters (**3a** and **3b**) from 4-trifluoromethoxy benzoyl chloride (**2a)**, 2,4,6-trichlorobenzoyl chloride (**3b)** respectively were synthesized through a 1:1 ratio, with that being cholesterol (0.5 mmol) and the aroyl chloride (0.5 mmol). (**3c**, **3d**, **3e**, and **3g**) were synthesized through a 1:2 ratio (cholesterol (0.5 mmol) and aroyl chloride (1.0 mmol)). Lastly, (**3f**) was synthesized through a 4:1 ratio making cholesterol 2.0 mmol and the aroyl chloride 0.5 mmol. The overall goal of this research is to synthesize cholesterol Esters by developing a cross-coupling chemistry process, while being able to see the broader application of those new types of cholesterol Esters in chemical biology. This is of importance because the formulation of these compounds is also valuable in drug discovery, due to cholesterol esters’ significant role in balancing the homeostasis within an individual’s body and other biological systems.

## 3. Procedure

The synthesis of these newly composed compounds began with a clean vial to begin the process. For the cholesterol and aroyl chloride, a 1 to 2 ratio was used (0.5 mmol of the cholesterol and 1.0 mmol of the aroyl chloride). Cholesterol was quickly yet carefully added to the vial (0.5 mmol, 194.0 milligrams), along with the base sodium tert-butoxide (1.0 mmol, 100 mg). After the cholesterol and base are added, the particularly selected aroyl chloride for that experiment will be included. When adding the aroyl chloride, it is important to identify if the compound is in a liquid or solid state. This information is of importance because an argon flush will need to be completed for this reaction. So, for example, if the aroyl chloride 4-cyanobenzoyl chloride is added and it is solid at room temperature, we would be safe in continuing the experiment. However, if the aroyl chloride is in a liquid form, then the compound would need to be administered to the vial after the argon flush. The cholesterol, sodium tert-butoxide, aroyl chloride, and PdCl_2_ (d^t^bpf) are added into the clean vial. 1,4-dioxane is added into the vial through the septum via syringe. The resulting mixtures were irradiated at 100˚C for 2 hours. Concluding the microwave chemistry, the vial was taken and prepared to do a vacuum filtration by creating a celite bed in the sintered funnel. Attached to the bottom of the funnel was a flask to collect the compound. After the vacuum suction is attached to the funnel, the product mixture in vial gets poured into the funnel. Next, the filtration process is aided by pouring ethyl ether into the funnel. This helps the compound to flow from the funnel into the flask. Once about 100 mL of the compound and ethyl ether are in the flask, we then put the flask on the evaporator for about 30 minutes to an hour, or until the mixture within the flask has completely evaporated. The flask is taken from the evaporator machine, and 1 to 2 milliliters of chloroform-d are added into the flask to prepare for NMR reading.

## 4. Conclusion

The cross-coupling method can be used to synthesize a wide variety of cholesterol esters, including cholesterol esters with different fatty acid chains and cholesterol esters with different functional groups. This makes the cross-coupling method a versatile tool for the synthesis of cholesterol esters for a variety of applications. The newly developed cross-coupling process of making bulky cholesterol esters from cholesterol and aroyl chlorides is a new entity for this topic. These types of esters have potential biological activity. Further studies are underway.
